# Silencing BMI1 eliminates tumor formation of pediatric glioma CD133+ cells not by affecting known targets but by down-regulating a novel set of core genes

**DOI:** 10.1186/s40478-014-0160-4

**Published:** 2014-12-20

**Authors:** Patricia A Baxter, Qi Lin, Hua Mao, Mari Kogiso, Xiumei Zhao, Zhigang Liu, Yulun Huang, Horatiu Voicu, Sivashankarappa Gurusiddappa, Jack M Su, Adekunle M Adesina, Laszlo Perlaky, Robert C Dauser, Hon-chiu Eastwood Leung, Karin M Muraszko, Jason A Heth, Xing Fan, Ching C Lau, Tsz-Kwong Man, Murali Chintagumpala, Xiao-Nan Li

**Affiliations:** Laboratory of Molecular Neuro-Oncology, Baylor College of Medicine, Houston, TX 77030 USA; Texas Children’s Cancer Center, Baylor College of Medicine, Houston, TX 77030 USA; Department of Pathology, Texas Children’s Hospital, Baylor College of Medicine, Houston, TX 77030 USA; Department of Neurosurgery, Texas Children’s Hospital, Baylor College of Medicine, Houston, TX 77030 USA; Molecular and Cellular Biology, Dan L. Duncan Cancer Center, Baylor College of Medicine, Houston, TX 77030 USA; Department of Neurosurgery, University of Michigan Medical School, Ann Arbor, MI 48109 USA; Department of Cell and Development Biology, University of Michigan Medical School, Ann Arbor, MI 48109 USA

## Abstract

Clinical outcome of children with malignant glioma remains dismal. Here, we examined the role of over-expressed BMI1, a regulator of stem cell self-renewal, in sustaining tumor formation in pediatric glioma stem cells. Our investigation revealed BMI1 over-expression in 29 of 54 (53.7%) pediatric gliomas, 8 of 8 (100%) patient derived orthotopic xenograft (PDOX) mouse models, and in both CD133^+^ and CD133^−^ glioma cells. We demonstrated that lentiviral-shRNA mediated silencing of suppressed cell proliferation in vitro in cells derived from 3 independent PDOX models and eliminated tumor-forming capacity of CD133^+^ and CD133^−^ cells derived from 2 PDOX models in mouse brains. Gene expression profiling showed that most of the molecular targets of BMI1 ablation in CD133^+^ cells were different from that in CD133- cells. Importantly, we found that silencing BMI1 in CD133^+^ cells derived from 3 PDOX models did not affect most of the known genes previously associated with the activated BMI1, but modulated a novel set of core genes, including RPS6KA2, ALDH3A2, FMFB, DTL, API5, EIF4G2, KIF5c, LOC650152, C20ORF121, LOC203547, LOC653308, and LOC642489, to mediate the elimination of tumor formation. In summary, we identified the over-expressed BMI1 as a promising therapeutic target for glioma stem cells, and suggest that the signaling pathways associated with activated BMI1 in promoting tumor growth may be different from those induced by silencing BMI1 in blocking tumor formation. These findings highlighted the importance of careful re-analysis of the affected genes following the inhibition of abnormally activated oncogenic pathways to identify determinants that can potentially predict therapeutic efficacy.

**Electronic supplementary material:**

The online version of this article (doi:10.1186/s40478-014-0160-4) contains supplementary material, which is available to authorized users.

## Introduction

Tumors of the central nervous system are the second most common cancer in children. Glioblastoma multiforme (GBM) is one of the most malignant brain tumors that occur both in children and adults. The primary treatment for GBM is surgical resection followed by chemotherapy and radiotherapy [1,2]. Overall survival for pediatric GBM (pGBM) patients remains poor, with 5-year survival rates of <20% [1]. Even in long-term survival patients, many children are left with significant physical and neuropsychological sequelae caused by therapy-related toxicities. Better understanding of tumor biology is needed for the development of new and more effective therapies.

Recent isolation of cancer stem cells (CSCs), also termed tumor-initiating cells [3-8], has created a new conceptual model for examining tumorigenesis and treatment failure. CSCs were shown to be resistant to standard chemotherapies and/or radiotherapies, causing tumor recurrence [9-13]. Thus, they have to be eliminated to cure disease. Many of the fundamental properties of CSCs are shared with normal stem cells [14,15]. Among them, the capability of self-renewal [3,4,15] plays the most important role in sustaining tumor growth. Therefore, genes and genetic pathways promoting abnormal self-renewal in CSCs should be prioritized for therapeutic targeting.

BMI1, a member of the polycomb group gene family, is an important regulator of self-renewal of hematopoietic and neural stem cells [16-19]. Mouse *Bmi1* was initially identified as a collaborator of c-myc [20,21]; and down-regulates p16 (Ink4a) and p19 (Arf) [17,22]. Over-expression of BMI1 has been reported in many different human cancers, including medulloblastoma [23-25] and adult GBM [26-28]. High level of BMI1 is associated with medulloblastoma invasion [29] and is also considered to be a poor prognostic marker in multiple human cancers [30-34], and is significantly involved in chemoresistance and tumor recurrence [35-38]. An 11-gene signature associated with the activated BMI1 was identified, and it reliably predicated shorter interval to recurrence and poor prognosis in 11 types of human cancers [39].

Several studies have shown that BMI1 is indispensable for self-renewal of normal and cancer stem cells [16,23,27]. The expression status and the functional roles of BMI1 in pGBMs stem cells, however, remain unknown. Additionally, while the genes and pathways associated with over-expressed BMI1 have been frequently reported, little is known about the genetic changes after the high level expression of BMI1 is knocked down in CSCs. Specifically, it is still not clear if silencing the aberrantly activated BMI1 in CSCs will affect the known target genes to reverse the phenotype; or if a new set of genes will be regulated to mediate the biological changes. Because there is increasing interest in developing targeted therapies against BMI1 [40], and integrated genetic analysis have revealed key differences between pediatric and adult GBM [41-44], it is important to determine the role of BMI1 in pGBM CSCs.

In this study, we examined if BMI1 is over-expressed in pediatric gliomas of various pathologic grades and if the over-expression of BMI1 was replicated in our new panel of 8 patient tumor-derived orthotopic xenograft (**PDOX**) mouse models. Using this set of clinically relevant animal models, we further examined if BMI1 expression was restricted to CD133^+^ cells, and if silencing BMI1 would significantly suppress cell proliferation in vitro and eliminate tumor formation in vivo. To understand the underlying molecular mechanisms, we performed global gene expression profiling in paired CD133^+^ and CD133^−^ cells and examined if it was the known targets or some new genes that were critical to the biological changes induced by silencing BMI1.

## Materials and methods

### Pediatric glioma tumors

**Figure 1:**
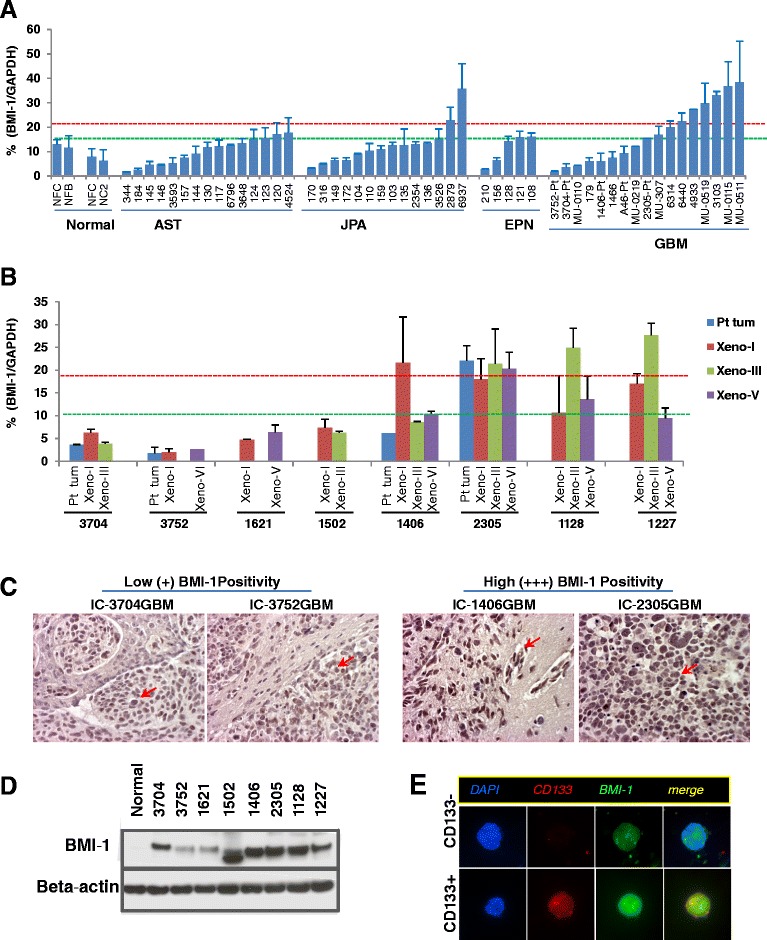
**Expression of BMI1 in pediatric gliomas. (A)** Analysis of BMI1 mRNA in pediatric brain astrocytomas (AST), juvenile pilocytic astrocytoma (JPA), ependymoma (EPN) and glioblastoma (GBM) using qRT-PCR. The relative levels of BMI1 were normalized to an internal control GAPDH and expressed as a percentage (mean ± SD). Red dotted lines indicate the levels of normal adult cerebral tissues. **(B)** BMI1 mRNA expression in the 8 PDOX mouse models. The relative levels of xenograft tumors during serial sub-transplantations from passage I to V (xeno-I to xeno-V) as well as the original patient tumors (Pt tum) (if available) were quantitated with qRT-PCR. **(C)** Representative immunohistochemical staining showing the GBM xenograft models with low-medium (IC-3704GBM and IC-3752GBM) and high BMI1 protein expressions (*arrows*) (IC-1406GBM and IC-2305GBM). **(D)** Western hybridization showing the increased BMI1 protein expression in the 8 GBM xenograft mouse models using beta-actin as loading control. Note that BMI1 protein was not expressed in the normal adult cerebral tissue, and the relative levels of BMI1 protein in the 8 xenograft models appeared to be correlated with their relative mRNA expressions, particularly in those with high mRNA transcripts (1406, 2305, 1128, and 1227). **(E)** Immunofluorescent staining showing that BMI1 protein was expressed in FACS-purified CD133+ and CD133- xenograft cells derived from ICb-1227AA.

Freshly resected pediatric brain tumor specimens were collected from 48 children undergoing surgery at Texas Children's Hospital, and 6 patients at the University of Michigan Medical School (Figure 1). Signed informed consent was obtained from the patient or their legal guardian prior to sample acquisition in accordance with local institutional review board (IRB) policy.

### Orthotopic transplantation into SCID mice

Surgical transplantation of tumor cells into mouse cerebrum and cerebellum was performed as we described previously [45,46], following the Institutional Animal Care and Use Committee (IACUC) approved protocol. Briefly, mice (aged 6–8 weeks of both sex) were anesthetized with sodium pentobarbital (50 mg/kg). Tumor cells (1 × 10^5^ for fresh surgical specimens, 1.5 × 10^3^ cells for CD133^+^ cells, and 1 × 10^4^ for CD133^−^ cells) were suspended in 2 μL of culture medium and injected into the cerebral hemisphere (1 mm to the right of the midline, 1.5 mm anterior to the lambdoid suture and 3 mm deep) via a 10-μL 26-gauge Hamilton Gastight 1701 syringe needle. Tumor cells from IC-1227AA, which was derived from a cerebellar anaplastic astrocytoma [45], were implanted to mouse cerebellar hemisphere (1 mm to the right of the midline, 1 mm posterior to lambdoid suture and 3 mm deep). The animals were monitored daily until they developed signs of neurological deficit or became moribund. They were then euthanized and their brains removed for histopathological analysis [45,46].

### Lenti-viral shRNA

Mission® shRNA Lentiviral transduction particles were obtained from Sigma-Aldrich. Five different Lentiviruses were obtained and screened for gene silencing efficacy. Listed below are the shRNA sequences. Lenti-BMI1-938: CCGGC GGAAA GTAAA CAAAG ACAAA CTCGA GTTTG TCTTT GTTTA CTTTC CGTTT TT; Lenti-BMI1-1061: CCGGC CTAAT ACTTT CCAGA TTGAT CTCGA GATCA ATCTG GAAAG TATTA GGTTT TT; Lenti-BMI1-693: CCGGC CAGAC CACTA CTGAA TATAA CTCGA GTTAT ATTCA GTAGT GGTCT GGTTT TT; Lenti-BMI1-1134: CCGGC CTACA TTTAT ACCTG GAGAA CTCGA GTTCT CCAGG TATAA ATGTA GGTTT TT; Lenti-BMI1-922: CCGGC CAGAA CAGAT TGGAT CGGAA CTCGA GTTCC GATCC AATCT GTTCT GGTTT TT. Lentiviruses expressing GFP (Lenti-GFP) were used to monitor transduction efficiency, and those containing no shRNA or non-target shRNA (Lenti-sh) were included as controls.

### Neurosphere assay

Xenograft tumor cells were incubated with serum-free media, containing Neurobasal medium, bFGF (50 ng/mL), EGF (40 ng/mL), N2, and B27 (Invitrogen) [45,46]. Lentiviruses carrying non-target shRNA or BMI1-specific shRNA were added to cell suspension (MOI =1). Cell proliferation was measured 2 weeks later with a cell counting kit (CCK) (Dojindo) as described previously [45,46].

### Western hybridization

Proteins were extracted from the xenograft tumors with Trizol (Invitrogen), and cells transduced with Lenti-shRNAs with Mammalian Protein Extraction agent (Piece) following the manufacturer’s instructions. Protein concentrations were assessed with the Bio-Rad DC protein assay kit. Equal amounts of protein were loaded onto Invitrogen 4-12% Bis-Tris gel. Proteins were then transferred onto PVDF membrane (Bio-Rad), blocked with 5% non-fat dry milk, and blotted with mouse monoclonal antibodies against BMI1-1 (05–637, MILLIPORE) and actin (3700S, Cell Signaling) followed by goat anti–mouse IgG-HRP (sc-2031, Santa Cruz) antibodies, respectively. The signals of actin and BMI1 were detected using ECL Substrate (Pierce).

### Quantitative real-time RT-PCR (qRT-PCR)

This was performed with SYBR green master mix in an ABI 7000 DNA detection system (ABI, Columbia, MD) as we previously described [47]. Complementary DNA was synthesized with MuLV reverse transcriptase and random hexamers (Perkin Elmer, Foster City, CA) in a total volume of 20 μl from 1 μg of total RNA extracted with TRIzol reagent (Invitrogen, Carlsbad, CA). Primers for PCR amplification were designed. Expression levels of the selected genes were normalized first to the internal standard GAPDH followed by normalization to lenti-non-target-shRNA using the standard ΔΔCt method [46]. All reactions were performed in duplicate and repeated twice. The specificities of PCR products were confirmed by analyzing dissociation curves from individual reactions and visualizations on 2% agarose gel.

### Immunohistochemical (IHC) Staining

IHC staining was performed using a Vectastain Elite kit (Vector Laboratories, Burlingame, CA) as described previously [48]. In brief, antigen retrieval was performed in a microwave oven in 0.03 M sodium citrate acid buffer. Endogenous peroxidase was quenched using hydrogen peroxide before sections were blocked in avidin D and biotin blocking reagent. After slides were incubated with monoclonal antibodies against BMI1-1 (MILLIPORE) (1:300) for 90 minutes at room temperature, the appropriate biotinylated secondary antibodies (1:200) were applied and incubated for 30 minutes, and the final signal was developed using the 3,3'-diaminobenzidine (DAB) substrate kit for peroxidase. For negative staining control, primary antibodies were replaced by PBS.

### Florescence activated cell sorting (FACS)

Xenograft tumor cells were incubated with monoclonal antibodies against CD133 that were conjugated with PE or APC at 4°C for 10 minutes, followed by flow sorting with MoFlo system. The dead cells were excluded with the use of propidium iodide (PI) staining.

### Immunofluorescence staining

The FACS-purified CD133^+^ and CD133^−^ cells were spread on positively charged slides, fixed with paraformaldehyde, and subjected to immunofluorescent staining using the Double Labeling Immunofluorescent Detection System (Lab Vision). Then, the antibodies against BMI1 and appropriate second antibodies were applied and detected with avidin conjugated with FITC. The images were captured with Pathvysion using a Nikon fluorescence microscope with a color CCD camera attached.

### Whole-genome gene expression profiling

The genome-wide expression analysis was performed using Illumina's Human-6 v2 BeadChips, which contains more than 48 K transcript probes, following manufacturer's instructions. Briefly, total RNA was extracted with TRIzol reagent (Invitrogen, Carlsbad, CA) as described previously [47]. RNA concentrations were measured using a Nanodrop 1000 spectrophotometer. RNA integrity was checked in electrophoresis using 2% agarose gel with 6% formaldehyde. A half-microgram of total RNAs was used to synthesize biotinylated cRNA using Totalprep RNA amplification kit (Ambion Inc, Austin, TX), and 1.5 μg of biotinylated cRNA was applied to the HumanWG-6 v2 Beadchips and processed according to the vendor’s instructions (Illumina, San Diego, CA). Each sample was loaded in duplicates on the chips. The Beadchips were scanned using a Beadstation 500 GX scanner.

The raw image files from the scanner were imported into the Bead Studio software Gene Expression module version 3.2.7 (Illumina Inc., San Diego, CA) and were processed using the quantile normalization algorithm. This method assumed that the distribution of the expression values did not change dramatically between arrays. All arrays were adjusted so that they showed an almost identical intensity distribution from all the genes. All 42620 elements in the gene profile tab in Bead Studio were used in the normalization. The log intensity values were analyzed in Bioconductor [49].

The differentially expressed genes were identified by comparing the normalized signal intensities induced by Lenti-BMI1-693 and Lenti-BMI1-922 with those by the non-target Lenti-shRNA through paired Student *t* test. *P* <0.05 and log2 fold changes >0.5 or < −0.5 was set as the cutoff for differentially expressed genes. Those genes whose expression was altered by the non-target Lenti-shRNA (*P* <0.05 and log2 fold changes >0.5 or < −0.5 when compared with the untreated control) were subtracted from the list. Heatmaps of different sets of the differentially expressed genes were created using average Pearson correlation and average linkage method implemented by the Multi-Experiment Viewer.

### Statistical analysis

Analysis of animal survival times was performed using log-rank analysis followed by pair-wise comparisons with the Holm-Sidak method and graphed with SigmaPlot 11 (Systat Software, Inc., San Jose, CA).

## Results

### Elevated expression of BMI1 mRNA in pediatric malignant gliomas

To determine the mRNA expression of BMI1 in pediatric gliomas, we applied qRT-PCR in a panel of 54 pediatric gliomas. Due to the difficulties of obtaining age-matched normal human cerebral tissues, normal RNA from 2 fetal brains, 1 adult cerebrum, and pooled RNA from 10 normal cerebrums (Clontech) were included as controls. The expression levels of BMI1 mRNA were normalized to that of GAPDH [46]. As expected, the BMI1/GAPDH ratio was higher in the fetal brains (12.3 ± 0.9%) than that in the adult cerebral tissues (7.1 ± 1.16%). When the BMI1/GAPDH levels in the pediatric brain tumors were normalized with that of the normal adult cerebral tissues, elevated BMI1 expression (>1.5 fold of adult cerebral tissues) was observed in 8 of 15 (53.3%) astrocytomas (2.3 ± 0.3 folds), 8 of 14 (57.1%) juvenile pilocytic astrocytomas (JPAs) (2.4 ± 1.2 folds), 3 of 5 (60%) ependymomas (2.18 ± 1.5 folds), and 10 of 17 (58.8%) GBMs (3.55 ± 1.1 folds). Further comparison with the fetal brains identified 2 of the 14 (14.2%) JPAs and 7 of the 17 (41.2%) GBMs that exhibited increased BMI1 expression (>1.5 fold of that in the fetal brains), a phenomenon that was not found in any of the 15 astrocytomas or the 5 ependymomas (Figure 1A). Combined, these data suggested that over-expression of BMI1 mRNA occurred in ~50% of pediatric brain tumors (compared with normal adult cerebral tissues), and pGBMs tend to have higher levels than that in the low-grade gliomas.

### Preservation of BMI1 over-expression in PDOX mouse models

To test the functional roles of BMI1 in pGBM, it is desirable to have clinically relevant pGBM mouse models that over-express BMI1. We have previously shown that primary-tumor derived orthotopic xenograft mouse models replicated the histology, invasive growth, and genetic abnormalities of pediatric brain tumors [45,50]. To further determine if BMI1 expression was replicated, we examined BMI1 mRNA expression in a panel of 8 patient-tumor derived pGBM xenograft models (Table 1), which were established through direct implantation of surgical specimens to the matched locations in mouse brains and strictly subtransplanted in vivo the brains of SCID mice [45,50]. In 4 of the 8 models in which the original patient tumors were available, the BMI1 expression was also examined. Elevated BMI1 mRNA expression (>1.5 folds of normal adult cerebral tissues) was observed in 4 models (IC-1406GBM, IC2305GBM, IC1128GBM and ICb-1227AA); whereas in the remaining 4 models (IC-3704GBM, IC-3752GBM, IC-1621GBM and IC-1502GBM), low levels of BMI1 mRNA transcripts were observed (Figure 1B). In all 8 models, relatively stable levels of BMI1 mRNA expressions were observed during serial sub-transplantations (from passage I up to V).

**Table 1:** **List of patient tumor-derived orthotopic xenograft mouse models**

**Model ID**	**Age**	**Gender**	**Diagnosis**	**BMI1**	**Ref**
IC-3704GBM	12 y	M	GBM (small cell variant)	+	[50]
IC-3752GBM	5 y	F	GBM	+	[50]
IC-1621GBM	6 y	M	GBM	+	[50]
IC-1502GBM	4 y 8 mo	F	GBM (giant cell )	+	[50]
IC-1406GBM	5 y	F	GBM	++	[50]
IC-2305GBM	9 y	M	GBM (small cell variant)	+++	[50]
IC-1128GBM	8 y 7 mo	M	GBM	++	[45]
ICb-1227AA	16 y 11 mo	F	Anaplastic Astrocytoma-(radiation induced)	++	[45]

Note: **IC:** intracerebral; **ICb**: intracerebellar.

To further estimate the relative abundance of BMI1 positive tumor cells, we performed IHC staining. In 4 models (IC-3704GBM, IC-3752GBM, IC-1621GBM, and IC-1502GBM), low (+) to medium (++) level positivity was detected, whereas in the remaining 4 models (IC-1406GBM, IC-2305GBM, IC-1128GBM and ICb-1227AA), strong (+++) BMI1 expression was observed (Figure 1C). In all these models, positive reaction was noted in >95% of tumor cells. Subsequent Western hybridization confirmed the elevated BMI1 protein expression in all xenograft models as compared with a normal cerebral tissue (obtained from a warm autopsy of an 8-year-old child) that lacked BMI1 expression (Figure 1D). The overall findings in Western blot correlated well with the results obtained from IHC staining, i.e. the xenografts with low (+) immunoreactivity were found to have weaker bands and those with high (+++) reactivity exhibited stronger bands. The significance of the smaller molecular weight band found in IC-1502GBM remains to be determined.

### BMI1 expression is not restricted to CD133^+^ cells

To determine if the expression of BMI1 protein is restricted to CSCs, we isolated CD133^+^ cells, the most commonly used marker to isolate GBM stem cells despite ongoing controversies [3,51-55], from GBM xenograft tumors and compared their BMI1 protein expression with CD133^−^ tumor cells. In both CD133^+^ and CD133^−^ cells derived from 7 GBM xenograft models, nuclear BMI1 expressions were observed (Figure 1E), suggesting that BMI1 over-expression is not restricted to CD133^+^ cells.

### Silencing BMI1 expression impairs self-renewal growth of glioma cells in vitro

**Figure 2:**
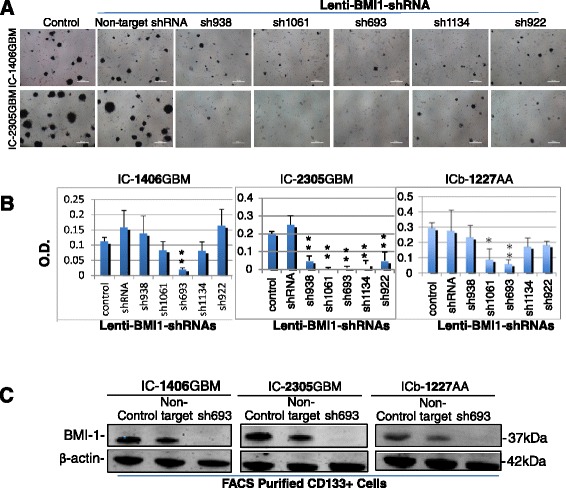
**Suppression of neurosphere formation in vitro in pGBM cells by lentivirus-mediated shRNA specific to BMI1. (A)** Representative images showing the suppression of neurosphere formation in two pGBM xenograft models (IC-1206GBM and IC-2305GBM). MTT was added 4 hr prior to the microscopic examination. Dark-colored intracellular crystal was indicative of viable cells. **(B)** Graphs showing the quantitative analysis of cell proliferation (** P < 0.01) in neurospheres derived from 3 independent PDOX models following the silencing of BMI1 with a panel of five lentivirus-mediated gene-specific shRNAs. O.D. indicates optical density. **(C)** Western hybridization showing the decreased BMI1 protein expression. CD133+ cells were purified from 3 PDOX models and transduced with Lenti-BMI1-693 (sh693) for 48 hr before being harvested for protein extraction. Tumor cells not treated with lentivirus (Control) and transduced with Lentiviruses containing non-target shRNA (Non-target) were included as references.

To evaluate the impact of silencing BMI1 expression on CSC self-renewal, changes of neurosphere forming efficiency in vitro in serum-free media containing EGF and bFGF, which is known to favor the growth of CSCs [45], were evaluated in three models (IC-1406GBM, IC-2305GBM and ICb-1227AA) that exhibited high levels of endogenous BMI1. The expression of BMI1 was silenced using a set of 5 lentiviral shRNAs that were specifically designed to knock down human BMI1. Microscopic examination revealed significant reduction of neurospheres in the cells transduced with Lenti-BMI1 shRNA (MOI = 1) (Figure 2A) as compared with the untreated cells and cells transduced with non-target shRNA (as mock control). Subsequent quantitation of cell viability identified Lenti-693 as the shRNA that produced most significant suppression of cell proliferation in all three models (Figure 2B) (*P* <0.01).

To further confirm that the suppressed cell proliferation was correlated with the silencing of BMI1 gene expression, changes of BMI1 protein were examined 48 hr post-lentiviral transduction in CD133^+^ cells purified with FACS from xenograft tumors (MOI =1). Compared with the cells transduced with the non-target shRNA that exhibited similar levels of BMI1 as the untreated cells, cells transduced with Lenti-BMI1-693 showed remarkable reduction of BMI1 protein expression in all three models (Figure 2C).

### Silencing BMI1-abrogated orthotopic xenograft tumor formation

**Figure 3:**
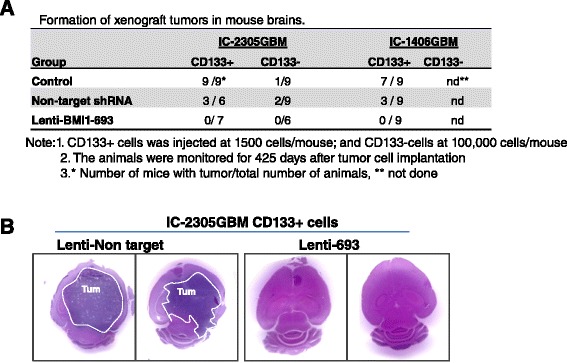
**Elimination of tumor-forming capabilities of CD133+ cells in vivo in mouse brains by Lenti-BMI1-693. (A)** Summary of tumor formation efficiency in tumor cells derived from the two pGBM mouse models. For CD133^+^ cells, each mouse was implanted with 1,500 cells; and for the CD133^−^ cells, 100,000 cells/mouse. **(B)** Representative images of H&E-stained paraffin sections. Compared with huge intracerebral xenografts in IC-2305GBM cells transduced with non-target Lenti-shRNA, no tumor formation was observed in mice implanted with tumor CD133+ cells in which BMI1 gene expression was silenced by Lenti-BMI1-693.

Previous studies have shown that BMI1 is critical in maintaining self-renewal of neural stem cells [16-18]. To determine the role of BMI1 in vivo in pGBM stem cells, we used ABC-conjugated antibodies against human CD133, the most common cell surface marker of human glioma stem cells despite the ongoing controversies [4,45,52-54,56-58], to purify CD133^+^ cells from IC-2305GBM (the more responsive model) and IC-1406GBM (the lest responsive model of the three) xenografts. After these cells were incubated with Lenti-BMI1-693 (MOI =1) for 48 hr (24 hr in serum-free media followed by an additional 24 hr selection in media containing puromycin), they were transplanted into the right cerebral hemisphere of NOD SCID mice (1,500 cells/mouse). The mice were monitored for up to 425 days. To confirm tumor growth, whole mouse brains were paraffin embedded, serially sectioned, and stained with hematoxylin-eosin. Compared with the tumor formation of 33.3% (3 of 9 mice) and 50% (3 of 6 mice) in the non-target shRNA groups (CD133 + ^Lenti-non-target^) from IC-1406GBM and IC-2305GBM, respectively, mice receiving the injection of CD133 + ^Lenti-BMI1–693^ cells did not form any tumors (0 of 9 mice in IC-1406GBM, and 0 of 7 mice in IC-2305GBM) (Figure 3A, B). Injection of CD133 + ^Lenti-BMI1–922^ cells resulted in tumor formation of 22.2% (2 of 9 mice) in the IC-1406GBM group and 0% (0 of 7 mice) in IC-2305GBM, which is in agreement with our findings in vitro in the cultured neurospheres (Figure 2A, B). These data suggested that BMI1 is required for the tumor formation of CD133^+^ pGBM cells in both models.

Since there are studies suggesting that not all glioma stem cells are CD133^+^ and our data indicated that BMI1 was also strongly expressed in CD133^−^ pGBM cells, we next examined if CD133^−^ pGBM cells were tumorigenic and if they also depended on BMI1 for tumor formation. In cells derived from IC-2305GBM, tumor formation was confirmed in 1 of 9 mice receiving untreated CD133^−^ cells, and 2 of 9 mice receiving CD133- ^Lenti-non-target^. Since the injected cell number of CD133^−^ (100,000/mouse) was far greater than that of the CD133^+^ cells (1,500/mouse), this result indicated that the tumor-forming capacity of CD133^−^ cells was lower than that of the CD133^+^ cells. Silencing BMI1 with Lenti-BMI1-693 and −922 led to complete abrogation of tumor formation in 6 and 7 mice, respectively. CD133^−^ cells from IC-1406GBM were not tested due to poor tumor-forming capacity. Combined, our data demonstrated that silencing the over-expressed BMI1 abrogated the tumorigenic capacity of CD133^+^ cells, highlighting BMI1 as a potential therapeutic target for pGBM CSCs.

### Understanding the mechanisms of action through gene expression profiling

To understand the mechanism with which silenced BMI1 block tumor formation and to identify new downstream targets, we performed global gene expression profiling in paired CD133^+^ and CD133^−^ cells derived from 3 models (IC-1406GBM, IC-2305GBM and ICb-1227AA) that expressed high levels of BMI1 at 48 hrs post lentiviral transduction. For each sample, RNAs from 3 sets of cells, 1) untreated control, and cell transduced with 2) non-target Lenti-shRNA, 3) Lenti-BMI1-693 were extracted and hybridized in duplicate on to Illumina arrays that contained 48 K elements. To identify the differentially expressed genes caused specifically by Lenti-BMI1-693, genes induced by the non-target Lenti-shRNA (*P* <0.05 and log2 fold changes >0.5 or < −0.5 when compared with the untreated control) were subtracted. The overall signal intensities of CD133^−^ cells derived from IC-2305GBM were too low to pass the quality control. This sample was excluded from the subsequent analysis.

**Figure 4:**
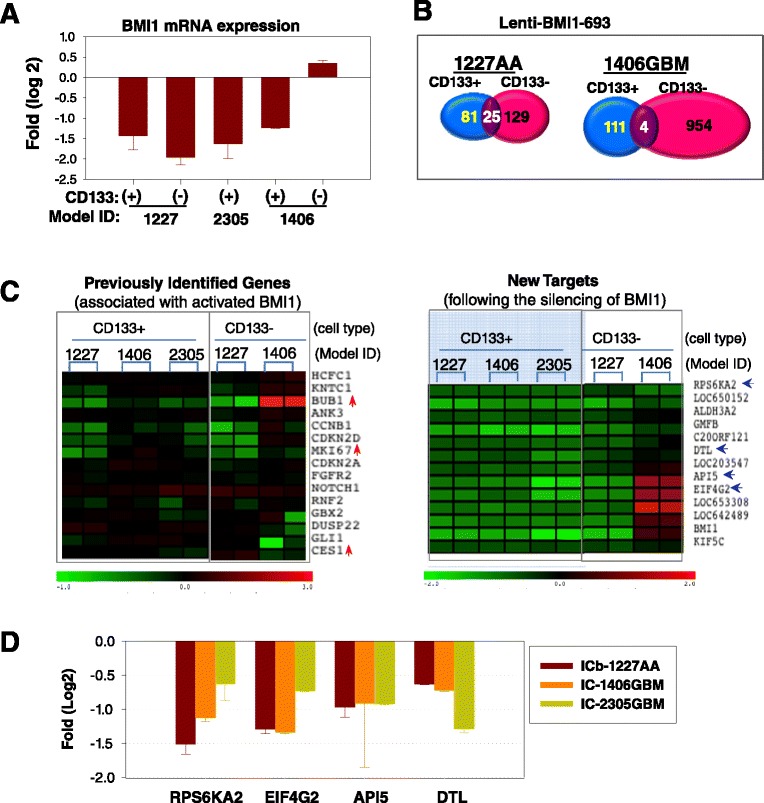
**Gene expression profiling in pGBM cells.** FACS-purified CD133^+^ and CD133^−^ cells were transduced with Lenti-BMI1-693 for 48 hr in vitro and harvested for RNA extraction. Tumor cells receiving no or non-target lenti-shRNA were included as control. **(A)** Down-regulation of BMI1 mRNA in CD133+ *(+)* and CD133- *(−)* cells transduced by Lenti-BMI1-693. For IC-2305GBM, the CD133^−^ cells were not included due to low cell content. **(B)** List of gene numbers differentially induced by lentin-BMI1-693 in CD133^+^ and CD133^−^ cells derived from two PDOX models. Only a small fraction of genes were shared. **(C)** Heatmaps of gene expression showing changes of known genes previously associated with the over-expressed BMI1 (*left panel*) and the new targets associated with the silencing of BMI1 (*right panel*). In the known gene panel, those genes originally up-regulated by over-expressed BMI1 are indicated with up-arrows (↑), and those inhibited by the over-expressed BMI1 are unmarked. No reversal of expression levels were observed in these genes. In the new target panel, genes consistently altered by Lenti-BMI1-693 in the CD133+ cells from all 3 models are highlighted in the blue shaded area. Arrows indicate the 4 genes that were validated with qRT-PCR in **(D)** where the mRNA levels in cells transduced with Lenti-BMI1-693 were normalized with those transduced with Lenti-non-target shRNA and plotted as fold changes (log2).

We first examined if BMI1 mRNA expression itself was affected. When compared with the cells transduced with the non-target Lenti-shRNA, the mRNA levels of BMI1 were significantly suppressed by Lenti-BMI1-693 in CD133^+^ cells from all three 3 models, and the CD133^−^ cells from ICb-1227AA. The CD133^−^ cells from IC-1406GBM were the only cells in which the BMI1 mRNA was not reduced by Lenti-BMI1-693 (Figure 4A). These results confirmed the silencing effects of Lenti-BMI1-693 on BMI1 expressions in CD133^+^ cells, and showed that that CD133^+^ and CD133^−^ glioma cells may not have equal responses toward BMI-1 silencing.

### Silencing BMI1 affected different sets of genes in CD133^+^ and CD133^−^ cells

Given that BMI1 was over-expressed in both CD133^+^ and CD133^−^ cells, we next examined if silencing BMI1 would affect the same downstream target genes (Figure 4B). In ICb-1227AA cells transduced by Lenti-BMI1-693, there were only 25 shared genes, representing 30.8% of 81 differentially expressed genes in CD133^+^ cells and 19.3% of 129 genes in CD133^−^ cells; whereas in IC-1406GBM, it was 4 of the 111 (3.6%) and 954 (0.4%) genes in CD133^+^ and CD133^−^ cells, respectively. Findings in IC-1406GBM CD133^−^ cells had to be interpreted with caution as BMI1 expression was not significantly suppressed by Lenti-BMI1-693 (Figure 4A). Nonetheless, these results demonstrated that silencing the over-expressed BMI1 affected different genes in CD133^+^ and CD133^−^ cells, suggesting that future evaluation of anti-BMI1 therapies should take into account the biological differences between CSC (e.g., CD133^+^ cells) and non-stem (e.g., CD133^−^) tumor cells.

### Silencing BMI1 did not affect the known target genes associated with the activated BMI1

We next examined if known BMI1 target genes were affected by silencing BMI1. Mice lacking *Bmi1* showed induction of both *p16Ink4a* and *p19Arf* in various hematopoietic and neuronal tissues [16]. As shown in Figure 4C, the mRNA levels of CDKN2A (INK4A, P16) and CDKN2D (INK4D, P19) were not affected, which was in agreement with previous findings in adult GBM [28,59]. We also examined Notch1, Gli1, and the 11 gene signature that was previously identified as associated with the over-expressed BMI1 gene in human malignant cancers [39] (Figure 4C). In CD133^+^ cells, silencing BMI1 did not significantly alter the expression of 3 genes (ANK3, FGFR2 and CES1) that were down-regulated by over-expressed BMI1, nor the 8 genes that were up-regulated by activated BMI1 [39] in any of the 3 GBM models. In CD133^−^ cells, BUB1 was the only gene whose expression was significantly up-regulated (>2 in log2 scale, *P* <0.05) in IC-1406GBM (Figure 4C). However, this gene was originally up-regulated by activated BMI1 and should have been suppressed. These data indicated that the genes associated with the over-expressed BMI1 were not reversed following the silencing of BMI1.

### Core gene expression signatures identified for CD133+ glioma cells

To identify new target genes affected by the silenced BMI1 in CD133^+^ glioma cells, we performed a series of pair-wise comparisons among the 3 models. Our goal was to identify the core-signature genes that were common to CD133^+^ cells. Our comparison of gene expression profiles of CD133^+^ cells identified a set of 12 genes (excluding BMI1) that were significantly down-regulated (P < 0.05 when compared with Lenti-shRNA, and log2 fold changes < −0.5) in all 3 models by Lenti-BMI1-693. These included RPS6KA2 (ribosomal protein S6 kinase, 90 kDa, polypeptide 2), API5 (apoptosis inhibitor 5), EIF4G2 (eukaryotic translation initiation factor 4 gamma, 2), DTL (denticleless E3 ubiquitin protein ligase homolog), KIF5C (kinesin family member 5C), ALDH3A2 (aldehyde dehydrogenase 3 family, member A2), GMFB (glia maturation factor, beta), and 5 less-known genes (LOC650512, LOC642489, LOC653308, LOC203547, and C20ORF121) (Figure 4C). Subsequent validation with qRT-PCR confirmed the down-regulation of RP6KA2, EIF4G2, API5, and DTL in the cultured CD133^+^ cells of these models (Figure 4D). Since none of the aforementioned genes had been previously identified with BMI1 signaling pathways, our study unraveled a set of genes that can potentially be the novel target genes in the BMI1 regulatory network in pGBMs. No similar genes were identified in the CD133^−^ cells derived from the 2 models, presumably because BMI1 was silenced only in the CD133^−^ cells in IC-1227AA.

**Figure 5:**
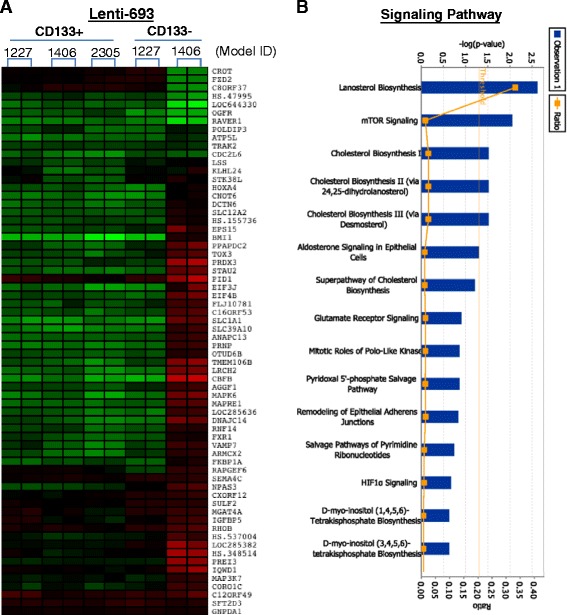
**Genes differentially altered by lenti-BMI1-693 in CD133** ^**+**^ **cells in at least 2 of the 3 PDOX derived cells. (A)** Heatmap showing the 46 genes in CD133+ cells as compared with the CD133^−^ cells. **(B)** Graph showing the top signaling pathways affected by the silencing of BMI1 in CD133^+^ cells.

There were an additional 46 genes induced by Lenti-BMI1-693 in CD133^+^ cells derived from 2 of the 3 glioma models (Figure 5A). Signaling pathway analysis identified lanosterol biosynthesis, mTOR signaling, and cholesterol biosynthesis as the top three pathways affected by Lenti-BMI1-693 (Figure 5B). Because the number of input genes was relatively low, more studies are needed to examine the significance of these and the remaining pathways, such as HIF1α and mitochondria dysfunction, in the Lenti-BMI1-693 group.

## Discussion

In this study, we confirmed the over-expression of BMI1 in a subset of pediatric gliomas, and showed that the levels of BMI1 mRNA correlated with tumor grade. We also demonstrated that BMI1 over-expression was maintained in a panel of 8 PDOX mouse models. More importantly, we demonstrated that silencing BMI1 expression in two independent pGBM models not only led to significant suppression of cell proliferation and neurosphere formation in vitro, but also caused the abrogation of xenograft tumor formation in vivo in mouse brains. Our global gene expression profiling further showed that silencing BMI1 in the CD133^+^ cells of pGBM did not affect the known target genes of the activated BMI1, and identified a novel set of core genes, including STAU2, RPS6KA2, ALDH3A2, FMFB, DTL, API5, EIF4G2, KIF5c, LOC650152, C20ORF121, LOC203547, LOC653308, and LOC642489, as potential downstream targets of the silenced BMI1 in pGBM.

To gain a relatively accurate estimate of BMI1 gene expression in pGBM, comparison with age-matched normal human brain tissue is highly desirable. Recognizing the difficulties of obtaining normal brain tissue from children, we utilized the normal fetal brains and normal adult cerebral RNAs that were commercially available as references. Although the total number of pediatric gliomas that over-expressed BMI1 mRNA varied depending on the normal references (fetal brain vs. adult brain), the trend remained the same, i.e. high-grade gliomas expressed higher levels of BMI1 mRNA than did low-grade gliomas. These results, combined with our findings in the PDOX mouse models, suggest that BMI1 over-expression might have played an important role in sustaining tumor growth of high-grade gliomas.

Cancer stem cell hypothesis suggests that only CSCs have the exclusive self-renewal capacity to form new tumors [3,60,61]. Since BMI1 is an established regulator of stem cell self-renewal, we focused our studies on CD133^+^ cells. This is because CD133 remains the most commonly used human glioma stem cell marker and has been successfully applied for the identification and isolation of glioma CSCs, despite ongoing controversies about the existence of CD133^−^ glioma stem cells [3,4,45,52-54,56-58] and it role in predicting glioma prognosis and survival [62-64]. We showed that silencing BMI1 with Lenti-BMI-693 completely eliminated tumor formation of the CD133^+^ cells (1,500/mouse) derived from IC-1406GBM and IC-2305GBM models that over-expressed BMI1. The overall activities of Lenti-BMI1-693 were the strongest, as it is the only lenti-BMI1-shRNA that suppressed cell proliferation in all three glioma models. Altogether, our data demonstrate that silencing BMI1 is sufficient to eliminate the tumor-forming capacity of pGBM stem cells in vivo.

Understanding the molecular mechanisms through which silencing BMI1 led to tumor abrogation is important for future development of targeted therapies. With pair-wise comparison of the whole genome gene expression profiles in FACS fractionated CD133^+^ and CD133^−^ tumor cells, we showed that CD133^+^ and CD133^−^ cells had distinct responsive genes following the silencing of BMI1. Identification of such differences between the matched CD133^+^ and CD133^−^ cells is important, as it not only confirms the biological differences between CSC and non-stem cancer cells, it also suggest that future evaluation of CSC associated genes/pathways should be performed in the appropriate cellular types, and data obtained from bulky tissues should be interpreted with caution.

It was also surprising to find that most of the known target genes previously associated with activated (over-expressed) BMI1, such as INK4A and ARF and the 11-gene signature, were not significantly altered. Although this result might have been caused by the biological differences between pGBM and adult cancers or by the pGBM CSC properties, our intriguing finding may also reflect the complex nature of gene expression regulations in human cancers. It suggested that the gene expression changes induced by the activated/over-expressed BMI1 did not have to alter again if the activated BMI1 is silenced. Confirmation of this phenomenon in additional human cancers should impact future development of targeted therapies, as the examination of known “target” genes - that have previously been associated with the overexpressed/activated therapeutic target - are frequently used as additional indicators or diagnostic markers of biological responses.

Our identification of a small set of 13 recurrent genes provided novel mechanisms through which silencing BMI1 inhibited pGBM CD133^+^ cells. These genes were shared by CD133^+^ cells derived from all 3 PDOX mouse models, and many of them have functions critical to the suppression of tumor formation. For example, down-regulation of the anti-apoptosis gene API-5 [65-68] may help to alleviate the anti-apoptosis activities; and inhibition of EIF4G2 [69] can potentially reverse its suppression of translation. Inhibition of glia maturation factor (GMFB) [70,71] and ribosomal protein S6 kinase (RPS6KA2) [72,73] may be implicated in controlling cell proliferation and differentiation.

## Conclusions

In summary, we demonstrated that BMI1 is over-expressed in pediatric gliomas, particularly in high-grade malignant gliomas, in both CD133^+^ and CD133^−^ cells; and silencing this gene with lentivirus-mediated shRNA led to elimination of the tumor-forming capacity, particularly of CD133^+^ tumor cells, in vivo in two independent and patient-specific PDOX mouse models, thereby supporting the development of new targeted therapies against BMI1 in pGBM. Furthermore, we identified a novel panel of 13 genes that were altered after silencing BMI1, highlighting the importance of re-analysis of the affected genes following the targeted therapy to identify the potential determinants and predicators of efficacy.

## Abbreviations

AA: Anaplastic astrocytoma
CSC: Cancer stem cell
GBM: Glioblastoma multiforme
CNS: Central nervous system
SCID: Severe combined immunodeficiency
Lenti-sh: Non-target lentiviral shRNA control
Lenti-BMI1-693: BMI1 specific lentiviral sRNA-693
Lenti-BMI1-922: BMI1 specific lentiviral shRNA-922
ICb: Intracerebellum
IC: Intracerebrum
IHC: Immunohistochemical
